# The Plant Invertase/Pectin Methylesterase Inhibitor Superfamily

**DOI:** 10.3389/fpls.2022.863892

**Published:** 2022-03-25

**Authors:** Daniele Coculo, Vincenzo Lionetti

**Affiliations:** Dipartimento di Biologia e Biotecnologie “C. Darwin”, Sapienza Università di Roma, Rome, Italy

**Keywords:** pectin methylesterase inhibitors, invertase inhibitors, sucrose metabolism, CW integrity, degree of methylesterification, plant growth and defence, biotechnological applications

## Abstract

Invertases (INVs) and pectin methylesterases (PMEs) are essential enzymes coordinating carbohydrate metabolism, stress responses, and sugar signaling. INVs catalyzes the cleavage of sucrose into glucose and fructose, exerting a pivotal role in sucrose metabolism, cellulose biosynthesis, nitrogen uptake, reactive oxygen species scavenging as well as osmotic stress adaptation. PMEs exert a dynamic control of pectin methylesterification to manage cell adhesion, cell wall porosity, and elasticity, as well as perception and signaling of stresses. INV and PME activities can be regulated by specific proteinaceous inhibitors, named INV inhibitors (INVIs) and PME Inhibitors (PMEIs). Despite targeting different enzymes, INVIs and PMEIs belong to the same large protein family named “Plant Invertase/Pectin Methylesterase Inhibitor Superfamily.” INVIs and PMEIs, while showing a low aa sequence identity, they share several structural properties. The two inhibitors showed mainly alpha-helices in their secondary structure and both form a non-covalent 1:1 complex with their enzymatic counterpart. Some PMEI members are organized in a gene cluster with specific PMEs. Although the most important physiological information was obtained in *Arabidopsis thaliana*, there are now several characterized INVI/PMEIs in different plant species. This review provides an integrated and updated overview of this fascinating superfamily, from the specific activity of characterized isoforms to their specific functions in plant physiology. We also highlight INVI/PMEIs as biotechnological tools to control different aspects of plant growth and defense. Some isoforms are discussed in view of their potential applications to improve industrial processes. A review of the nomenclature of some isoforms is carried out to eliminate confusion about the identity and the names of some INVI/PMEI member. Open questions, shortcoming, and opportunities for future research are also presented.

## Introduction

**Figure 1:**
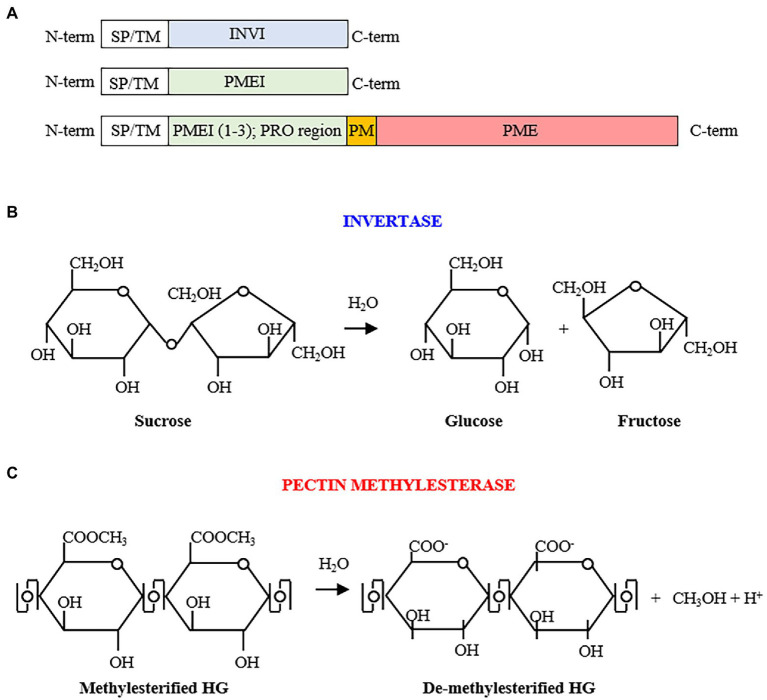
**(A)** Invertase inhibitor (INVI) and pectin methylesterase inhibitor (PMEI) structural organizations. The INVI/PMEIs domain are preceded by a signal peptide (SP) or a transmembrane domain (TM) for the targeting to the endomembrane system leading to the different subcellular localization. PMEI/INVIs can possess one, both, and neither of these motifs. Some PMEIs (from 1 to 3 isoforms; also named PRO region) can be clustered with a C-terminal PME. **(B)** Sucrose hydrolysis by invertase activity yielding glucose and fructose. **(C)** De-methylesterification of homogalacturonan (HG) by pectin methylesterases, with consequent production of negatively charged carboxyl groups, methanol, and protons.

**Figure 2:**
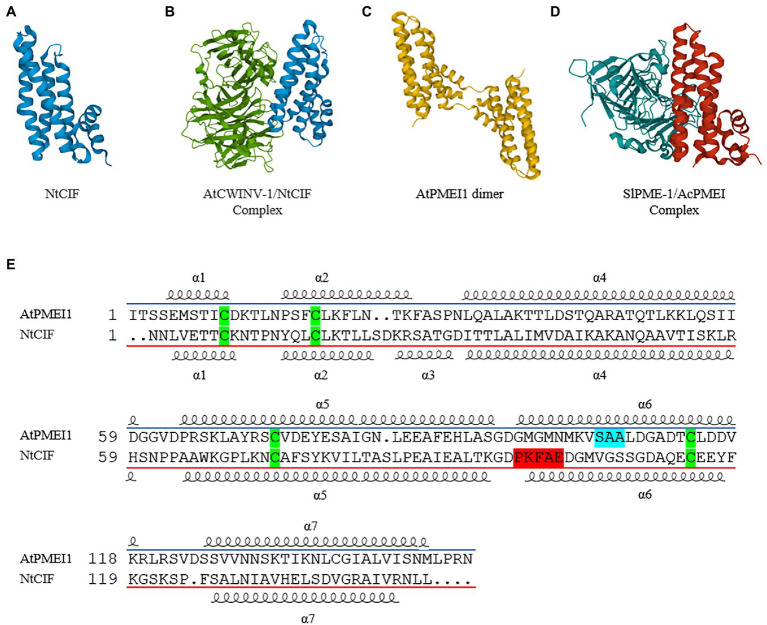
Crystal structure of different INVI/PMEI isoforms alone or in complex with their enzymatic counterpart. **(A)** NtCIF (PDB ID; 1RJ1) and **(B)** NtCIF in complex with AtCWINV-1 (PDB ID; 2XQR). **(C)** AtPMEI1 dimer (PDB ID; IX8Z) and **(D)** AcPMEI in complex with SlPME-1 (PDB ID; 1Xg2). **(E)** Sequence Comparison of AtPMEI1 and NtCIF. The most conserved motifs are highlighted in light blue for PMEI and in red for INVI. The four cysteine conserved in both inhibitors are highlighted in green. The *α* symbols followed by numbers indicates the different alpha-helices.

**Figure 3:**
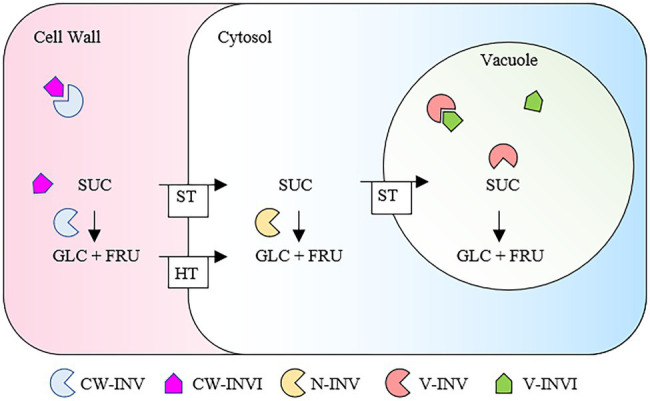
Subcellular localization and activities of INVs and INVIs. Sucrose can be cleaved in the apoplast by a CW-INV in glucose and fructose, which are transported into the cytoplasm by a hexose transporter. Sucrose can also be directly loaded by specific sucrose transporters into the cytosol or in the vacuole where it is cleaved by neutral invertase (N-INV; or sucrose synthase) or V-INV, respectively. The hexoses generated by INV activity can serve as substrate for growth as well as can regulate gene expression during growth and defense. Modulations of INVs by INVIs are dependent from different environmental stimuli and can influence different physiological processes. FRU, fructose; glc, glucose; SUC, Sucrose. ST, Sucrose Transporter; HT, Hexose Transporter.

Plant Invertase/Pectin Methyl Esterase Inhibitors (INV/PMEIs; PF04043)^1^ belong to a large protein superfamily acting in the tight post-transcriptional regulation of Invertases (INVs) and Pectin methylesterases (PMEs), two classes of enzymes with distinct enzymatic activities in carbohydrate metabolism (Figure 1; Gough et al., 2001). INVI/PMEIs are highly represented in different plant species (Table 1). Plant INVs (also known as *β*-fructosidases), convert the sucrose into its building blocks, fructose and glucose, central molecules for carbohydrate translocation, metabolism, and sensing in higher plants (Figure 1B; Roitsch and González, 2004). INVs play different roles in organ development, carbohydrate partitioning, sugar signaling, and response to biotic and abiotic stresses (Ruan et al., 2010; Tauzin and Giardina, 2014; Liao et al., 2020). Acid INVs and neutral/alkaline INVs were identified, showing different pH optima and subcellular compartments. The Acid INVs, belonging to gH32 (glycoside hydrolase family 32),^2^ shows an optimum pH of 3.5–5.0 and can be divided in cell wall (CW) and vacuolar (V) INVs. Neutral/alkaline INVs show an optimum pH of 6.8–9.0, belong to gH100, and appear to be localized to the cytosol, mitochondrion, plastids, and nucleus. The INV activity can be post-transcriptionally controlled by INV Inhibitor (INVI; Figures 1A, 2A,B, 3). Based on the subcellular site where their activity is exerted, CW- and V-INVIs (previously named also Inhibitor of *β*-Fructosidases; C/VIFs) can be distinguished (Rausch and Greiner, 2004; Figure 3). The first INVI was described more than 40 years ago in potato plants (*Solanum tuberosum*; Pressey, 1966). Although INVIs have been reported for various plant species, little is known about their roles in plant physiology (Bate et al., 2004; Raiola et al., 2004; Reca et al., 2008; Zhang et al., 2010; Lionetti et al., 2015b).

**Table 1:** INVI/PMEIs assignments in different plant species genomes.

Species	Common name	Number of proteins
*Actinidia chinensis Hongyang*		109
*Aegilops tauschii*		94
*Amborella trichopoda*		40
*Aquilegia coerulea*		63
*Arabidopsis lyrata*	Lyrate rockcress	132
*Arabidopsis thaliana*	Thale cress	125
*Brachypodium distachyon*	Stiff brome	60
*Brassica rapa*	Field mustard	167
*Capsella rubella*		130
*Carica papaya*	Papaya	54
*Citrus clementina*		75
*Citrus sinensis*	Sweet orange	78
*Cucumis sativus*	Cucumber	62
*Eucalyptus grandis*	Rose gum	46
*Fragaria vesca*	Wild strawberry	82
*Glycine max*	Soybean	163
*Gossypium raimondii*		152
*Hordeum vulgare*	Domesticated barley	68
*Linum usitatissimum*	Flax	160
*Lotus japonicus*		69
*Malus domestica*	Apple	144
*Manihot esculenta*	Cassava	100
*Medicago truncatula*	Barrel medic	171
*Mimulus guttatus*	Spotted monkey flower	115
*Musa acuminata*	Wild Malaysian banana	58
*Musa balbisiana*	Balbis banana	88
*Nicotiana benthamiana*		160
*Oryza sativa*	Rice	81
*Panicum virgatum*	Switchgrass	131
*Phaseolus vulgaris*	French bean	104
*Phoenix dactylifera*	Date palm	13
*Phyllostachys heterocyclavar*	Kikko-chiku	30
*Physcomitrella patens*		12
*Picea abies*	Norway spruce	53
*Picea sitchensis*	Sitka spruce	5
*Pinus taeda*	Loblolly pine	82
*Populus trichocarpa*	Black cottonwood	118
*Prunus persica*	Peach	70
*Ricinus communis*	Castor bean	71
*Selaginella moellendorffii*		13
*Setaria italica*	Foxtail millet	67
*Solanum lycopersicum*	Tomato	86
*Solanum pimpinellifolium*	Currant tomato	86
*Solanum tuberosum*	Potato	113
*Sorghum bicolor*	Sorghum	73
*Thellungiella halophila*		105
*Theobroma cacao*	Cacao	72
*Triticum aestivum*	Bread wheat	95
*Triticum urartu*		85
*Vitis vinifera*	Wine grape	21
*Zea mays*	Maize	76
*Zea mays*	Maize	79

Source: https://supfam.mrc-lmb.cam.ac.uk/SUPERFAMILY/index.html

**Figure 4:**
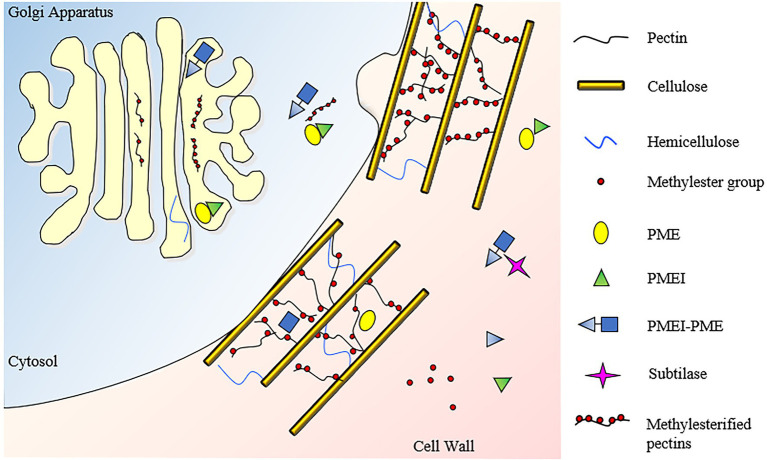
Subcellular localization and activities of PMEI-PMEs and PMEIs. HG is methylesterified in the golgi apparatus, where PMEIs can avoid a premature pectin de-methylesterification by PME, which could cause a pectin jellification. Pectin is secreted in the apoplast in a high methylesterified form. In this compartment, a fine-tuning of PME activity is exerted by independent and clustered PMEIs to regulate the degree and pattern of pectin methylesterification in various plant physiology processes. Subtilisin-like proteases (Subtilases) can degrade the processing motif of PMEI-PME catalyzing the separation of the inhibitor from the PME domain.

A member of INVI/PMEI family can be classified as PME Inhibitor (PMEI) if it is able to inhibit a PME activity (Figures 1A–C, 2C,D, 4). The first PMEI, named AcPMEI, was discovered in ripe fruit of kiwi (*Actinidia chinensis*; Balestrieri et al., 1990). PMEs (CE8, Carbohydrate Esterase) catalyze the de-methylesterification of pectin, releasing free carboxyl ester groups, protons, and methanol (Figure 1C). PMEs are encoded by large multigene families in many plant species (Pelloux et al., 2007; Harholt et al., 2010). Until now, these enzymes were linked to the modulation of the degree and pattern of methylesterification of homogalacturonan (HG), the major component of pectin secreted in a highly methylesterified form to the CW (Figure 4). The degree of methylesterification constitutes an important factor influencing stiffness and hydration status of the pectic matrix (Catoire et al., 1998; Willats et al., 2001). The current knowledge on the mode of de-methylesterification of the single plant PME isoforms remains scarce. The existence of different methylester distributions on HG *in vivo* suggests the involvement of multiple PME isoforms with different action patterns. A blockwise de-methylesterification results in the production of adjacent free galacturonic acid units that can form calcium crosslinks between HG chains, known as “egg-box” structures, resulting in pectin stiffening (Limberg et al., 2000; Wu et al., 2018). Instead, the random de-methylesterification results in the removal of one methylester group at a time from various non-contiguous residues on the HG chains exposing the polymer to the activity of pectinolytic enzymes (Limberg et al., 2000). While this latter mechanism has been demonstrated for PMEs of microbial origin, plant PMEs with a random de-methylesterification have not been identified so far. PME isoforms finely tune the degree and pattern of methylesterification during multiple developmental processes, such as stomata function (Amsbury et al., 2016; Huang et al., 2017), cell adhesion (Lionetti et al., 2014a; Daher and Braybrook, 2015), organ development, and phyllotactic patterning (Peaucelle et al., 2011b; Senechal et al., 2014). Plant PMEs also play a critical role in multiple plant–microbe interactions and stress responses (Lionetti et al., 2012). An immunity triggered PME activity, driven by specific PME isoforms, is exploited against pathogens (Bethke et al., 2014; Del Corpo et al., 2020). This activity is triggered to modulate pectin methylesterification in *Arabidopsis thaliana* against fungi, such as *Botrytis cinerea* and *Alternaria brassicicola*, bacteria, such as *Pseudomonas syringae*, and viruses like *turnip vein clearing virus* (*TVCV*; Bethke et al., 2014; Lionetti et al., 2014b, 2015a, 2017). Moreover, PMEI activity and pectin methylesterification status play important roles during plant resistance to abiotic stresses (Wu et al., 2018).

A PMEI can be transcribed independently or in pairs with a Type I PME as one polycistronic messenger RNA which resembles an operon-like gene cluster (Figure 1A; Boycheva et al., 2014). In these PMEI-PMEs clusters, PMEI region (also referred to as PRO region, PRO domain, or PMEI-like region) can be separated from the PME domain by specific subtilisin-like serine proteases (subtilases) before excretion of PME domain or later into the apoplast during different physiological processes (Figueiredo et al., 2018; Schaller et al., 2018). Different evidence indicates that in these clusters, PMEI domain acts as an intramolecular inhibitor of PME enzymatic activity (Bosch et al., 2005; Wolf et al., 2009; Del Corpo et al., 2020). Other works indicate that PMEI domain can be required for the targeting of PMEs toward the CW (Wolf et al., 2009) or that it could work as intramolecular chaperone in the regulation of PME folding (Micheli, 2001). Further investigation is needed to understand the subcellular action of both independent and PME-clustered PMEIs.

Gene structure and sequence analyses show that the origin of the independently expressed PMEIs may be derived from the neofunctionalization of the PMEI domain from the PMEI-PME genes (Wang et al., 2013). PMEI-PME clusters evolved during the divergence of moss from charophytes, while independent PMEIs appear later in land plants. The physiological reasons which prompted plants to initially evolve a coordinated expression of the enzyme with its inhibitor counterpart and, later, to involve also a PME inhibition using independent PMEIs, deserves further investigation. Most likely, both PMEI-PMEs clusters and independent PMEIs were fundamental factors finely tuning the pectin methyl-esterification in the CW remodeling that emerged as necessary in land plants. The existence of specific pairs between PMEIs and PMEs has been hypothesized based on data obtained *in vitro* (Table 2). A single PMEI can inhibit multiple plant PMEs and the PMEI characterized up to now are unable to inhibit microbial PMEs (Lionetti, 2015; Lionetti et al., 2015b). The bacterial enzymes show much longer turns that protrude out of the *β*-helix making its putative active site cleft deeper and narrower than that of plant PMEs, a feature that could prevent the approach of the inhibitor to the active site of the enzyme (D’Avino et al., 2003).

**Table 2:** Arabidopsis INVI-PMEI independent protein isoforms.

	Gene ID	AGI code	Symbol	Possible interactor	Function	Literature
1	837879	At1g02550				
2	837458	At1g09360				
3	837459	At1g09370				
4	837620	At1g10770				
5	6240451	At1g11362				
6	7922417	At1g11593				
7	838054	At1g14890				
8	838929	At1g23205				
9	838944	At1g23350			Plant stresses	Coolen et al., 2019
10	841214	At1g47960	AtCW-INVI1, AtC/VIF1		Seed germination, Root length, Plant-pathogen interaction, Salt susceptibility	Link et al., 2004; Siemens et al., 2011; Su et al., 2016; Yang et al., 2020
11	841219	At1g48010				
12	841220	At1g48020	AtPMEI1	ATPPME1, AtPMEI-PME17, AtPMEI-PME3, AtPMEI-PME16	Plant growth, Pavement cells morphogenesis, Plant-pathogen interaction	Wolf et al., 2003; Raiola et al., 2004; Lionetti et al., 2007; Lin et al., 2021
13	6240492	At1g50325				
14	841456	At1g50340				
15	841904	At1g54620				
16	841939	At1g54980				
17	842026	At1g55770				
18	842062	At1g56100	AtPMEI14		Mucilage release	Shi et al., 2018; Ding et al., 2021
19	842117	At1g56620	AtPMEI16		Mucilage release	Shi et al., 2018; Ding et al., 2021
20	842370	At1g60760				
21	842574	At1g62760	AtPMEI10		Salt susceptibility, Plant-pathogen interaction,	Jithesh et al., 2012; Lionetti et al., 2017
22	842576	At1g62770	AtPMEI9	AtPME3	CW integrity, Root growth	Sorek et al., 2015; Hocq et al., 2017b
23	843391	At1g70540	EDA24		Embryo sac	Pagnussat et al., 2005
24	843409	At1g70720				
25	814690	At2g01610				
26	815562	At2g10970				
27	816026	At2g15345				
28	3768435	At2g31425				
29	817701	At2g31430	AtPMEI5		Seed germination, Seedling emergence, Plant growth	Wolf et al., 2012; Müller et al., 2013; Jonsson et al., 2021
30	6241279	At2g31432				
31	819319	At2g47050				
32	819347	At2g47340				
33	819380	At2g47670	AtPMEI6		Mucilage release	Saez-Aguayo et al., 2013
34	3768856	At3g05741	AtPMEI15		Mucilage release	Shi et al., 2018; Ding et al., 2021
35	820471	At3g12880				
36	820970	At3g17130	AtPMEI8		CW integrity, Root growth	Sorek et al., 2015, p. 16
37	820971	At3g17140				
38	820972	At3g17150				
49	5008004	At3g17152				
40	820981	At3g17220	AtPMEI2	ATPPME1, AtPMEI-PME3, AtPMEI-PME16	Plant growth, Plant-pathogen interaction	Wolf et al., 2003; Raiola et al., 2004; Tian et al., 2006; Lionetti et al., 2007
41	820982	At3g17225				
42	28719277	At3g17227				
43	820983	At3g17230				
44	6240965	At3g27999				
45	819850	At3g36659				
46	823892	At3g47380	AtPMEI11		Plant-pathogen interaction	Lionetti et al., 2017
47	823921	At3g47670				
48	824095	At3g49330				
49	824734	At3g55680				
50	825391	At3g62180				
51	825457	At3g62820				
52	828192	At4g00080	UNE11		Embryo sac	Pagnussat et al., 2005
53	7922364	At4g00872				
54	827589	At4g02250				
55	828628	At4g25250	AtPMEI4	AtPMEI-PME3; AtPMEI-PME17	Root growth	Pelletier et al., 2010
56	6240679	At4g03945				
57	827253	At4g15750	AtPMEI13		Mucilage release	Shi et al., 2018; Ding et al., 2021
58	828566	At4g24640	APPB1			Holmes-Davis et al., 2005
59	828628	At4g25250				
60	828629	At4g25260	AtPMEI7	AtPMEI-PME3		Sénéchal et al., 2015
61	832197	At5g20740	AtPMEI3		Phyllotaxis, Rhyzotaxis, Pavement cells morphogenesis	Peaucelle et al., 2008; Haas et al., 2020; Wachsman et al., 2020
62	832508	At5g24370				
63	833851	At5g38610				
64	834739	At5g46930				
65	834740	At5g46940				
66	834741	At5g46950	InvINH2			Zuma et al., 2018
67	834742	At5g46960	AtPMEI12		Plant-pathogen interaction	Lionetti et al., 2017; Zuma et al., 2018
68	834743	At5g46970				
69	834744	At5g46980				
70	834745	At5g46990				
71	835067	At5g50030				
72	835068	At5g50040				
73	835069	At5g50050				
74	835070	At5g50060				
75	835071	At5g50070				
76	835226	At5g51520				
77	836355	At5g62340				
78	836356	At5g62350				
79	836357	At5g62360	AtPMEI17		Salt and Aphid tolerance, Freezing susceptibility	Chen et al., 2018; Silva-Sanzana et al., 2019
80	836583	At5g64620	AtCW/V-INVI2; AtC/VIF2		Plant-pathogen interaction	Link et al., 2004; Siemens et al., 2011

From a structural point of view, PMEIs and INVIs, despite having a low aa sequence identity (20–30%), they share several structural properties. The three-dimensional structures of AtPMEI1 from Arabidopsis and of a CW-INVI from tobacco (*Nicotiana tabacum*; NtCIF) were elucidated (Hothorn et al., 2004a,b; Figures 2A,C). Both fold in a four-helix bundle structure preceded by an N-terminal extension thought to play an important role during the enzymatic inhibition. Both inhibitors hold four conserved cysteine residues typically engaged in the formation of two disulfide bridges, important to stabilize both two *α* helices of the hairpin loop and two α helices of the four-helical bundle structure. The structure of AtPMEI1 (Figure 2C) is composed of a four-helix bundle that arranges the helical components in an up-down–up-down topology with disulfide bridges. The N-terminal region, composed of two short and distorted helices, extends outside the central domain, forming a hairpin. The orientation of this hairpin allows the extensive contacts with the α-hairpin of a neighboring molecule forming an active dimer in solution. The N-terminal region of AtPMEI1 was proposed to be crucial for the interaction with a PME from Carrot (*Daucus carota*; Hothorn et al., 2004b). Although this model does not fit with the crystallographic data regarding the complex between AcPMEI and SlPME-1 of tomato (*Solanum lycopercsicum*; Figure 2D; Di Matteo et al., 2005), where the N-terminal region of AcPMEI does not establish contact with PME, it cannot be excluded that the two inhibitors use two different modes of interaction. AcPMEI interacts with PME at the level of the active site by forming a stoichiometric 1:1 complex in which the inhibitor covers the shallow cleft of the enzyme where the putative active site is located (Di Matteo et al., 2005; Ciardiello et al., 2008). The four-helix bundle of AcPMEI packs roughly perpendicular to the parallel *β*-helix of SlPME-1, and three of these helices (and not an helix of the N-terminal extension as proposed for AtPMEI1) interact with SlPME-1 in proximity of the putative active site (Di Matteo et al., 2005). The crystal structure of the complex between the AtCWINV-1 from Arabidopsis and the NtCIF was also elucidated (Figures 2A,B; Hothorn et al., 2010). The structure revealed that the four-helix bundle of NtCIF binds primarily in the substrate-binding cleft of the five-bladed β-propeller module of invertase. PME and INV activities and their complexes with their respective inhibitors are pH sensitive (Hothorn et al., 2010; Bonavita et al., 2016; Hocq et al., 2017b). There is no information available on the three-dimensional structure of PMEI-PME clusters.

A PMEI or an INVI can hold specific features in their amino acid sequence (Figure 2E). PMEI has a conserved Threonine residue, previously demonstrated to strengthen the interaction with PME at the acidic apoplastic pH, a typical Serine, Alanine, Alanine (SAA) amino acid motif in α6 helix, and a C-terminal hydrophobic region of six amino acids involved in the stabilization of the four-helical bundle structure of the protein (Di Matteo et al., 2005). Instead, the amino acid motif Proline, Lysine, Phenylalanine (PKF) in α6 helix, as well as the contiguous Alanine and glutamic Acid (AE) residues are the sequence fingerprints highly conserved in INVIs and critical for INVI-INV interaction. Both INVIs residues, important for the formation of the complex with INVs, and the distortion of INVI α2 helix could be responsible for the lack of interaction between INVI and PME (Di Matteo et al., 2005). Unfortunately, these features do not always help to understand the identity of an isoform. For example, PKF motif is absent in AtC/VIF2 (Link et al., 2004).

## The Arabidopsis INVI/PMEI Superfamily

Most of the knowledge on the role of INVI/PMEIs in plant physiology has so far been gained by studying *A. thaliana*, the annual dicotyledonous plant, served as a model for physiological studies in many laboratories. For this reason, this review will deal specifically with all Arabidopsis isoforms, integrating them with data obtained in other species. We identified *in silico* 125 INVI/PMEIs in Arabidopsis, 80 independent INVI/PMEIs, and 45 PMEI-PME clusters (Tables 1–3). Given the need to verify the type of inhibitory activity, the INVI-PMEIs members cannot be pre-numbered as for other families. The name of various members was assigned by the scientific community based on the chronological order they were characterized. However, confusion about the identity and the names of some INVI/PMEI member begins to appear in literature. For example, both PMEI and INVI functions have been proposed for the same AgI code. At5g46960, although named *InvINH1,* its INVI activity has never been demonstrated (Zuma et al., 2018). Rather, At5g46960 is the *AtPMEI12*, which possess the SAA amino acid motif and influences PME activity, the degree of pectin methylesterification, and the HG integrity in Arabidopsis during *B. cinerea* infection (Lionetti et al., 2017). Also, two different isoforms were named with the same name. This is the case of At4g15750 and At5g62360 isoforms both called *AtPMEI13* by different authors (Chen et al., 2018; Shi et al., 2018; Silva-Sanzana et al., 2019). To avoid confusion and respecting the chronology of publications, we rename At5g62360 as *AtPMEI17*. Moreover, the lack of symbol for At3g60730 and At2g26450 led us to name them, respectively, *AtPMEI-PME65* and *AtPMEI-PME66*. Also unfortunately, the AtPMEI-PME17 isoform was considered an independent PMEI in different papers (Leyva-González et al., 2012; Takahashi et al., 2019). We propose to rename Type I PMEs, by adding the PMEI-tag in front of PME (PMEI-PMEs; Table 3) to allow the scientific community to immediately recognize a PMEI co-expressed with a PME. We will begin to discuss the findings on PMEIs, given the greater amount of data available compared to INVIs.

**Table 3:** Arabidopsis INVI-PMEI protein isoforms clustered with a PME.

	Gene ID	AGI code	PME Symbol	New symbol	Function	Paper
1	838078	At1g02810	AtPME7	AtPMEI-PME7	Probable pseudogene	Dedeurwaerder et al., 2008
2	837701	At1g11580	AtPME18; AtPME-PCRA	AtPMEI-PME18	Root growth, Plant- pathogen interaction	Micheli et al., 1998, p. 3; De-la-Peña et al., 2008; Lionetti et al., 2017; Stefanowicz et al., 2021
3	837702	At1g11590	AtPME19	AtPMEI-PME19		
4	838928	At1g23200	AtPME6, HIgHLY METHYL ESTERIFIED SEEDS (HMS)	AtPMEI-PME6	Stomatal function, Embryo development, Mucilage release	Levesque-Tremblay et al., 2015; Amsbury et al., 2016
5	841820	At1g53830	AtPME2	AtPMEI-PME2	Callus formation	Xu et al., 2018
6	841821	At1g53840	AtPME1	AtPMEI-PME1		
7	817184	At2g26440	AtPME12	AtPMEI-PME12		
8	817185	At2g26450	No number	AtPMEI-PME66		
9	818907	At2g43050	AtPME16; ATPMEPCRD	AtPMEI-PME16		
10	819130	At2g45220	AtPME17	AtPMEI-PME17	Root growth, Plant-pathogen interaction	Senechal et al., 2014; Del Corpo et al., 2020
11	819317	At2g47030	AtPME4; VgDH1	AtPMEI-PME4	Pollen tube growth	Jiang et al., 2005
12	819318	At2g47040	AtPME5; VgD1	AtPMEI-PME5	Pollen tube growth	Jiang et al., 2005
13	819368	At2g47550	AtPME20	AtPMEI-PME20		
14	819727	At3g05610	AtPME21	AtPMEI-PME21		
15	819728	At3g05620	AtPME22	AtPMEI-PME22		
16	819867	At3g06830	AtPME23	AtPMEI-PME23		
17	820240	At3g10710	AtPME24; RHS12	AtPMEI-PME24	Root hair development	Won et al., 2009; Cheong et al., 2019
18	820241	At3g10720	AtPME25	AtPMEI-PME25		
19	820650	At3g14300	AtPME26; ATPMEPCRC	AtPMEI-PME26		
20	820651	At3g14310	AtPME3	AtPMEI-PME3	Seed germination, Root development, Pavement cells morphogenesis, Plant-pathogen interactions, Metal tolerance	Micheli et al., 1998; Hewezi et al., 2008; Raiola et al., 2011; Weber et al., 2013; Guénin et al., 2017; Lin et al., 2021
21	822422	At3g27980	AtPME30	AtPMEI-PME30	Beneficial bacterial recruitment, Plant-pathogen interaction	Lakshmanan et al., 2013; Zehra et al., 2021
22	823402	At3g43270	AtPME32	AtPMEI-PME32		
23	823894	At3g47400	AtPME33	AtPMEI-PME33		
24	824083	At3g49220	AtPME34	AtPMEI-PME34	Transpiration, Heat tolerance	Huang et al., 2017; Wu et al., 2017
25	825070	At3g59010	AtPME35	AtPMEI-PME35	Mechanical strength of stem	Hongo et al., 2012, p. 35
26	825244	At3g60730	No number	AtPMEI-PME65		
27	825390	At3g62170	AtPME37; VgDH2	AtPMEI-PME37	Pollen tube growth	Jiang et al., 2005
28	828218	At4g00190	PME38	AtPMEI-PME38	Probable pseudogene	Dedeurwaerder et al., 2008
29	827708	At4g02300	AtPME39	AtPMEI-PME39		
30	828067	At4g02320	AtPME40	AtPMEI-PME40		
31	828064	At4g02330	AtPME41; ATPMEPCRB	AtPMEI-PME41	Chilling tolerance	Qu et al., 2011
32	825703	At4g03930	AtPME42	AtPMEI-PME42		
33	827282	At4g15980	AtPME43	AtPMEI-PME43		
34	829458	At4g33220	AtPME44	AtPMEI-PME44		
35	829459	At4g33230	AtPME45	AtPMEI-PME45		
36	830378	At5g04960	AtPME46	AtPMEI-PME46	Metal tolerance	Geng et al., 2017
37	830379	At5g04970	AtPME47	AtPMEI-PME47		
38	830836	At5g09760	AtPME51	AtPMEI-PME51		
39	832209	At5g20860	AtPME54	AtPMEI-PME54		
40	832850	At5g27870	AtPME28	AtPMEI-PME28		
41	834977	At5g49180	AtPME58	AtPMEI-PME58	Mucilage release	Turbant et al., 2016
42	835223	At5g51490	AtPME59	AtPMEI-PME59		
43	835224	At5g51500	AtPME60	AtPMEI-PME60		
44	835418	At5g53370	AtPME61; ATPMEPCRB	AtPMEI-PME61		
45	836585	At5g64640	AtPME64	AtPMEI-PME64		

## PME Inhibitors

**Figure 5:**
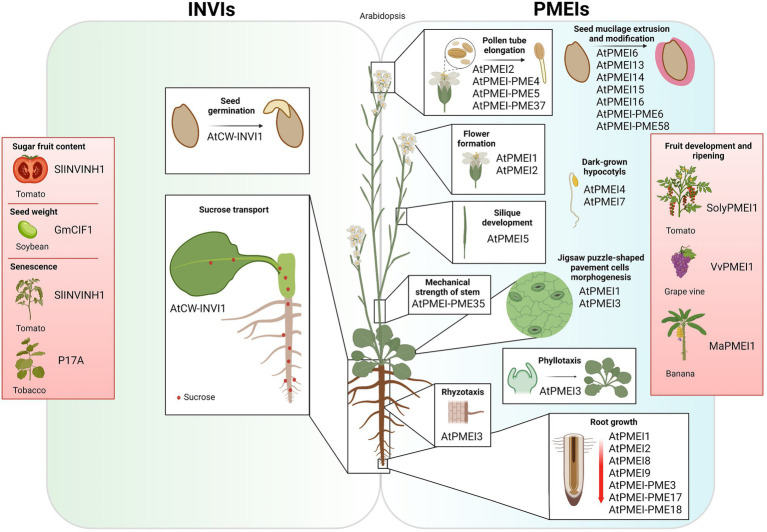
Overview of INVIs and PMEIs functions in plant growth and development. INVIs control seed germination, sugar transport in roots; senescence and sugar fruit content. PMEIs play multiple roles in several physiological processes, such as pollen tube elongation, seed mucilage extrusion, and modification, flowering transition, silique development, mechanical strength of stem, phyllotaxis, rhyzotaxis, pavement cells morphogenesis, hypocotyl growth in the dark, and root growth, and they are also involved in fruit development and ripening.

**Figure 6:**
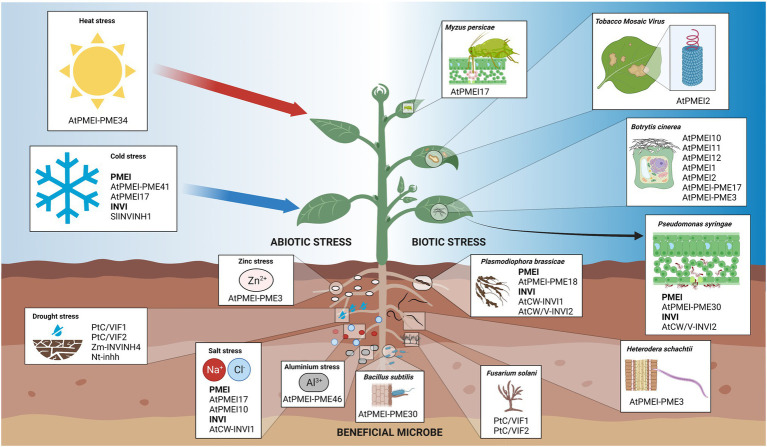
Schematic representation of the involvement of INVI/PMEI members in plant-environment interactions. Plants face different pathogens and pests, such as bacteria, nematodes, fungi, viruses, and insects as well as multiple abiotic stresses. Different INVI/PMEI members have a pivotal role plant-microbe associations beneficial to the host plant like also during multiple plant-pathogen interactions. PMEIs and INVIs are also involved in plant responsiveness to different abiotic stresses and in heavy metal tolerance.

To date, 17 INVI/PMEI isoforms were already identified as independent PMEI in Arabidopsis, although only AtPMEI1, AtPMEI2, and AtPMEI7 were purified and their activities verified. The inhibitory activity of the PMEI region in the PMEI-PME cluster was demonstrated for AtPMEI-PME17 (Del Corpo et al., 2020). The remaining isoforms were considered PMEIs because their transgenic overexpression or mutation leads to an alteration of PME activity and/or of the pectin methylesterification, with consequences in different plant physiology processes (Figures 5, 6). From here on, considerations on the role of PMEI-PMEs will be understood as actions of the enzyme controlled by the inhibitor in the specific cluster.

### PMEIs Assist Pollen Tube Growth During Fertilization and Embryogenesis

Pollen tube tip growth in the transmitting tract is crucial for reproductive success of plants. Pollen tube elongation is driven by secretion of pectic material and a gradient of degree of pectin methylesterification along the pollen tube axis provides the plasticity and rigidity requested for pollen tube growth (Parre and geitmann, 2005). PME and PMEI can play multiple roles in this process in different species (Bosch and Hepler, 2005; Zhang et al., 2010; Rocchi et al., 2011). AtPMEI1 and AtPMEI2 transcripts are particularly expressed in flower tissues suggesting a role of these inhibitors during flower formation or during the reproductive process. Both proteins interact with, and inhibit *in vitro*, different PMEs indicating a large spectrum of recognition for the PMEIs (Table 2; Raiola et al., 2004; Lionetti et al., 2007; Röckel et al., 2008). An interesting role in the regulation of the dynamics of pectin metabolism in polar pollen cell growth was demonstrated for AtPMEI2 (Röckel et al., 2008). AtPMEI2 showed a polarized accumulation at the pollen tube apex favored by a local PMEI endocytosis at the flanks of the tip. By reducing blockwise de-methylesterification and Ca^2+^-mediated pectin crosslinks, the localized AtPMEI2 accumulation at pollen tube apex favors CW extensibility and polar growth. Also, AtPMEI-PME5, AtPMEI-PME4, and AtPMEI-PME37 clusters are highly expressed during pollen tube growth (Jiang et al., 2005). A controlled AtPMEI-PME5 activity by PMEI domain could modulate pectin methylesterification of the transmitting tract cells to assist pollen tube movement toward the ovules.

The remodeling of pectin methylesterification play important roles in embryogenesis (Cruz-Valderrama et al., 2018; Pérez-Pérez et al., 2019). AtPMEI12 is expressed in the micropylar endosperm that surrounds the embryo and its prolonged expression suppressed embryo growth in Arabidopsis, most likely, by disturbing pectin methylesterification homeostasis (Zuma et al., 2018). AgL62 and FIS2 are required to regulate the expression of AtPMEI12 in the syncytial endosperm (Hoffmann et al., 2022). AtPMEI-PME6 is required for cell wall loosening in the embryo to facilitate cell expansion (Levesque-Tremblay et al., 2015).

### PMEIs Regulate Pectin Extensibility During Emergence, Formation, and Growth of Different Organs

Precise spatiotemporal modifications of CW composition and structure are critical for cell expansion and shape (Somerville et al., 2004). Pectin remodeling underlie changes in CW elasticity in organ initiation and differentiation (Hocq et al., 2017a; Shin et al., 2021). The phyllotaxis in the Arabidopsis shoot apical meristem is accompanied by a pectic de-methylesterification in subepidermal tissue layers which is strictly controlled by AtPMEI3 (Peaucelle et al., 2008, 2011a). The *AtPMEI3* overexpression in Arabidopsis produced, throughout the meristem dome, a significant reduction of “egg-box” structures, the development of shorter cells, and loss of growth asymmetry leading to an altered primordia outgrowth. Also in rhyzotaxis, AtPMEI3 expression, acting on pectin methylesterification, influences the functionality of the root clock for lateral root formation (Wachsman et al., 2020).

The jigsaw puzzle-shaped pavement cells of the leaf epidermis serve as an attractive model to investigate the mechanisms for cell–cell coordination of cell shapes (Yang, 2008). Pectin nanofilament expansion drives morphogenesis in plant epidermal cells (Haas et al., 2020). Plant overexpressing AtPMEI1 or AtPMEI3 showed defects in interdigitation and lobe formation in the pavement cells of the leaf epidermis indicating that the pectin methylesterification can influence plant cell morphogenesis (Haas et al., 2020; Lin et al., 2021). Auxin-induced callus formation is considered as a cell reprogramming process for *in vitro* regeneration of plants. Transgenic plants overexpressing AtPMEI-PME2 developed callus-like structures in the roots when grown on medium without exogenous auxin indicating that this cluster participates in the cell reprogramming during callus formation (Xu et al., 2018).

The Arabidopsis dark-grown hypocotyls and root growth were extensively used as models to study pectin modifications during organ elongation (Hocq et al., 2017b). The growth of hypocotyls in the dark is biphasic, with an initial slow and synchronous growth and a subsequent growth acceleration that propagates rapidly from the base to the top of the hypocotyls. AtPMEI4 controls the timing of the growth acceleration modulating the PME activity and pectin de-methylesterification (Pelletier et al., 2010). *AtPMEI4* overexpression showed an increased concentrations of methylesterified pectins and a delay of growth acceleration. AtPMEI7 was also detected in apoplastic proteins extracted from dark-grown hypocotyl although its role in this process remain to be demonstrated (Sénéchal et al., 2015).

Modulation of PMEIs expression can have different effects on root and root hair growth. A decrease in PME activity correlated with an increased root length in *atpmei-pme17* mutants and in plant overexpressing *AtPMEI1*, *AtPMEI2*, or *AtPMEI9* compared to controls (Lionetti et al., 2007, 2010; Wolf et al., 2012; Senechal et al., 2014, p. 17; Hocq et al., 2017b). On the contrary, a *pmei4* mutant and a AtPMEI-PME3 overexpressor, both expressing an elevated PME activity in Arabidopsis root CWs, showed an increased root length (Hewezi et al., 2008; Senechal et al., 2015). Specific PMEI-PME interactions and their regulation could underlie the contrasting root phenotypes observed in the transgenic plants. AtPMEI1 can inhibit the AtPMEI-PME17 activity *in vitro* (Del Corpo et al., 2020). AtPMEI7 expressed in *Escherichia coli* can form *in vitro* a pH-dependent reversible complex with AtPMEI-PME3 (Sénéchal et al., 2017). Indirect evidence indicates that AtPMEI4/AtPMEI-PME17, AtPMEI4/AtPMEI-PME3 and AtPMEI9/AtPMEI-PME3 interactions are likely to occur *in vivo* (Senechal et al., 2015; Hocq et al., 2017b). The inhibition capacity of AtPMEI4 was predicted to be highly pH-dependent, for the presence of key protonatable amino acids interacting with AtPMEI-PME3. *AtPMEI5* overexpression in Arabidopsis caused root waiving in seedlings and strong defects in adult plants like fusion of cauline leaf and shoot as well as strongly impaired silique development (Wolf et al., 2012; Müller et al., 2013). These effects were related to brassinosteroids as part of a compensatory response against the loss of CW integrity, triggered by an imbalance in pectin methylesterification. Moreover, the AtPMEI5 overexpressors germinate earlier and faster compared to control suggesting that pectin methylesterification is essential for the temporal regulation of radicle emergence in endospermic seeds by altering the mechanical properties of the CWs (Muller et al., 2013). Interestingly, *AtPMEI5* overexpression also revealed a correlation between HG methylesterification and auxin distribution in cell elongation that induce hypocotyl bending required for seedling emergence (Jonsson et al., 2021). AtPMEI8 and AtPMEI9 were identified during a suppressor screen for genetic suppressors of *cobra*, an Arabidopsis mutant with a reduced root length associated to defects in cellulose formation and an increased ratio of unesterified/esterified pectin (Sorek et al., 2015). *AtPMEI8* and *AtPMEI9* expression is exaggerated in mutants with CW defects. The overexpression of *AtPMEI8* and *AtPMEI9* increases the amount of pectin methylesterification in the *cob-6* mutant at the wild-type levels and partially restore the cobra root growth suggesting that pectin methylesterification is a significant factor for CW integrity. AtPMEI-PME3 is ubiquitous in Arabidopsis tissues and it was involved in multiple physiological processes like adventitious rooting, root hair production, and seed germination (Guénin et al., 2011, 2017). AtPMEI-PME24 plays a role in root hair development and it can be inhibited by the non-proteinaceous PME inhibitor phenylephrine (Won et al., 2009; Cheong et al., 2019).

Lignin in secondary CW is considered the main component influencing the mechanical strength of the stem. Interestingly, the de-methylesterification of the primary CW can play a role in CW stiffening for mechanical support of the Arabidopsis inflorescence stem (Hongo et al., 2012). A loss-of-function mutant of AtPMEI-PME35 showed a pendant stem phenotype and an increased deformation rate of the stem.

### PMEI in Fruit Development, Ripening, and Postharvest Fruit Processes

Fruit development and ripening require a combined, sequential, and synergistic action of a range of CW degrading enzymes (CWDEs; Wang et al., 2018). Advanced softening during ripening is a limiting factor in fruit shelf life and storage (Brummell and Harpster, 2001). The role of PME in fruit ripening was intensively examined in tomato. PME activity can affect pectin structure during ripening and fruit processing and it can also be a potential enhancers of ascorbic acid production (Tieman et al., 1992; Gaffe et al., 1994; Tieman and Handa, 1994; Thakur et al., 1996; Rigano et al., 2018). PMEIs can modulate PME activity and pectin methylesterification in different stages of fruit life. Several inhibitors were identified and characterized from the fruits of different species, like AcPMEI in Kiwi (Balestrieri et al., 1990), SolyPMEI1 in Tomato (Reca et al., 2012), MaPMEI1 in Banana (*Musa acuminata*; Srivastava et al., 2012) and VvPMEI1 in grapevine (*Vitis vinifera*; Lionetti et al., 2015b). The *AcPMEI*, *SolyPMEI,* and *MaPMEI1* expressions increase as the fruits ripen to finely control pectin de-methylesterification in softening during ripening. Differently, VvPMEI1 control PME activity at early phases of grape berry development to assist a rapid cell growth and to maintain pulp firmness, by preventing precocious pectin degradation and grape berry softening.

Different evidence indicates PMEI activity as a valid tool in food processes (Sørensen et al., 2004). PME is physiologically released into the juice during processing, and it is considered a juice clarifying enzyme. PME activity, by triggering the formation of “egg-box” structures, causes the precipitation of pectins and cloud loss in juice, one of the major problems in fruit juice manufacturing industries (Bazaraa et al., 2020). The thermal inhibition of PME activity might be a solution but it can negatively affect the nutritional quality of the juice. The addition of PMEI during the process was demonstrated to reduce phase separation improving juice quality (Castaldo et al., 1991; Sørensen et al., 2004; Bellincampi et al., 2005). PME activity can represent also a postharvest problem in grape fermentation and distillation processes, inducing a high methanol content in spirits (Botelho et al., 2020). PMEI can reduce methanol formation in grape must and marc as well as in products derived by fermentation and distillation (Lante et al., 2008; Zocca et al., 2008).

### PMEIs Modulate Seed Mucilage Extrusion and the Mucilage Degree of Methylesterification

The mucilage secretory cells present in the epidermal layer of the seed coat are responsible for mucilage production and release (Francoz et al., 2015). The release and function of mucilage are affected by PME activity (Rautengarten et al., 2008), and several evidence indicate that this activity requires a fine regulation by PMEIs. AtPMEI6 is specifically expressed in seed coat epidermal cells and *pmei6* mutants showed a delayed mucilage release (Saez-Aguayo et al., 2013). The analysis of PME activity in soluble mucilage from *pmei6* and 35S:PMEI6 transgenic plants indicates that AtPMEI6 inhibits endogenous PME activities. The level of *AtPMEI6* expression in transformants correlated with the level of methylesterified HG revealed using antibodies recognizing HG methylesterification status. This evidence leads to conclude that AtPMEI6 controls CW integrity of seed coat epidermal cells by preventing HG de-methylesterification for a correct seed mucilage release. Mechanistic insights indicate that the AtPMEI6-dependent partially methylesterified HG pattern represents an amphiphilic polysaccharidic platform necessary for PEROXIDASE36-specific anchoring, useful to loosen the outer periclinal wall domains of mucilage secretory cells, necessary for mucilage extrusion (Kunieda et al., 2013; Francoz et al., 2019).

AtPMEI13, AtPMEI14, AtPMEI15, and AtPMEI16 are other four independent mucilage-related PMEIs (Shi et al., 2018; Ding et al., 2021). *pmei13* and *pmei14* mutants but not *pmei15* mutant showed an increased PME activity and a reduced degree of methylesterification in the seed mucilage. AtPMEI15 might play only a minimal role in HG de-methylesterification or it could also be an INVI. AtPMEI14 protein seems dedicated to the modulation of pectin de-methylesterification in the mucilage after its release because any discernible mucilage extrusion defects were detected in the *pmei14* mutant. The expression of *AtPMEI6*, *AtPMEI14,* and *AtPMEI16*, like also other pectin modifying enzymes can be activated by the transcriptional factors GLABRA2 (GL2), *LEUNIG_HOMOLOG/MUCILAGE MODIFIED1* (*LUH/MUM1*), *SEEDSTICK* (*STK*), and MYELOBLASTOSIS 52 (MYB52; Saez-Aguayo et al., 2013; Ezquer et al., 2016; Ding et al., 2021). The Arabidopsis ETHYLENE RESPONSIVE ELEMENT BINDING FACTOR 4 (ERF4) and the MYB52 transcription factors interact and play antagonistic roles in the regulation of pectin de-methylesterification in seed mucilage. ERF4 directly suppresses the expression of *AtPMEI13*, *AtPMEI14*, *ATPMEI15* and suppresses *AtPMEI6* indirectly by antagonizing MYB52 function, giving rise to positive regulation of pectin de-methylesterification during seed development (Ding et al., 2021). Also the clusters AtPMEI-PME6 and AtPMEI-PME58 were associated to HG modification during mucilage release (Levesque-Tremblay et al., 2015; Turbant et al., 2016). AtPMEI-PME6 is required for mucilage extrusion while AtPMEI-PME58 activity participates in the regulation of interactions between HG and other polymers (probably rhamnogalacturonan I) during the formation of the mucilage adherent layer.

### PMEIs Are Involved in Multiple Biotic and Abiotic Stresses

A fine modulation of PME activity and pectin methylesterification is exerted to face biotic and abiotic stresses (Lionetti et al., 2012; Bellincampi et al., 2014). Plants involve a spatiotemporal modulation of PME activity against multiple pathogens to trigger defense response in several ways (Bethke et al., 2014; Lionetti, 2015; Del Corpo et al., 2020). PMEs can induce the formation of the “egg-box” structures, resulting in pectin stiffening (Limberg et al., 2000). Moreover, PME activity can favor the production of damage-associated molecular patterns. For instance, PMEs can promote the release or perception of de-methylesterified oligogalacturonides, able to trigger plant immunity (Osorio et al., 2008, 2011; Ferrari et al., 2013; Kohorn et al., 2014). De-methylesterification of pectin by PMEs can also generate the alarm signal methanol (Hann et al., 2014). Methanol and oligogalacturonides are able to trigger a defensive priming in plants (Dorokhov et al., 2012; Komarova et al., 2014; Gamir et al., 2021; Giovannoni et al., 2021). Moreover, the pathogen recognition receptors Wall Associated Kinase 1 (WAK1), WAK2, and FERONIA (FER) preferentially bind to de-methylesterified pectins (Decreux and Messiaen, 2005; Kohorn et al., 2014; Feng et al., 2018; Guo et al., 2018; Lin et al., 2021). Independent and clustered PMEIs were involved in plant immunity, at different times during microbial infection. Intriguingly, INVI/PMEIs families show gene duplications, which are frequent in stress-related genes and are beneficial for survival in challenging environments (Oh et al., 2012; Kalunke et al., 2015). *atpmei-pme17* mutants exhibited increased susceptibility to *B. cinerea* indicating that AtPMEI-PME17 cluster contributes to trigger PME activity against *B. cinerea* (Del Corpo et al., 2020). *AtPMEI-PME17* expression is regulated by defense signaling pathways suggesting its involvement for early defense response. At later stages of infection, an extensive PME mediated de-methylesterification of pectin could favor pectin degradation by microbial CWDE. This effect can be restrained by the expression of independent PMEIs (Lionetti et al., 2017). at*pmei10*, at*pmei11*, and at*pmei12* mutants showed increased PME activity, decreased pectin degree of methylesterification, and increased susceptibility to infection indicating that AtPMEI10, AtPMEI11, and AtPMEI12 can be exploited late during *Botrytis* infection to lock an extensive decrease of pectin methylesterification to defend pectin integrity. Intriguingly, the evidence that AtPMEI11 is induced by oligogalacturonides suggests a system of amplification of the pectin protection during immunity. Consistently, the overexpression of *AtPMEI1* and *AtPMEI2* in Arabidopsis induced a high degree of pectin methylesterification that correlated with a low susceptibility to *B. cinerea* (Lionetti et al., 2007). Similar results were obtained also in other pathosystems and also in monocots where the pectin level is low (An et al., 2008; Volpi et al., 2011; Liu et al., 2018). It must be emphasized that PMEI overexpression is free of disease resistance/developmental growth trade-offs observed in plants with engineered CWs (Pontiggia et al., 2020; Ha et al., 2021). Rather, PMEI overexpressors had a higher biomass yield and can improve tissue saccharification in bioconversion (Lionetti et al., 2010, 2014a; Francocci et al., 2013). Early aphid infestation induces an increase in PME activity, methanol emissions, and HG de-methylesterification (Silva-Sanzana et al., 2019). at*pmei17* mutants (named pmei13 mutants in the original article) are significantly more susceptible to the green peach aphid (*Myzus persicae*) compared to control in terms of settling preference, phloem access, and phloem sap drainage. AtPMEI17 seems particularly effective in plant–aphid interaction, since aphid feeding activities were not altered in AtPMEI6 overexpressors.

Induced Systemic Resistance (ISR) triggered by microbial bio-agents showed strong potential for biocontrol against phytopathogens (Zehra et al., 2021). Interestingly, the beneficial microbe *Bacillus subtilis* manipulate PME activity in root to improve own colonization while promoting plant resistance to leaf microbes (Lakshmanan et al., 2013). Roots of *atpmei-pme30* mutant inoculated with showed increased bacterial root colonization and foliar protection against the pathogen *P. syringae*.

Also, pathogens can exploit plant PMEI-PMEs to create an optimal cellular environment for their own survival. The ubiquitous AtPMEI-PME3 seems particularly targeted. AtPMEI-PME3 is exploited by *B. cinerea* and *Pectobacterium carotovorum* as susceptibility factor required for tissue degradation and colonization (Raiola et al., 2011). Cyst nematodes use own CWDEs, such as cellulases and pectinases to breach the CW for their root penetration and migration (Bohlmann and Sobczak, 2014). A cellulose binding domain-containing protein released by the sugar beet cyst nematode *Heterodera schachtii* into Arabidopsis tissues interacts with AtPMEI-PME3 to aid cyst nematode parasitism (Hewezi et al., 2008). PME activity, by reducing the level of pectin methylesterification can improve the accessibility of other CWDEs to CW polymers, assisting syncytium development. The cellulose binding domain-containing protein could interact with AtPMEI-PME3 in the cytoplasm followed by a potential joint export into the CW. The PMEI region of AtPMEI-PME3 could protect pectin in the golgi apparatus from premature de-methylesterification. CW remodeling of Arabidopsis root cells is also exploited by the obligate biotrophic *Plasmodiophora brassicae*, a protist pathogen that causes clubroot disease in brassica species (Stefanowicz et al., 2021). A pectin de-methylesterification mediated by AtPMEI-PME18 can favor the release of resting spores of the fungus. Intriguingly, AtPMEI-PME18 showed both PME and ribosome-inactivating proteins activity (De-la-Peña et al., 2008). Intriguingly, AtPMEI-PME18 activity could be manipulated by *P. brassicae* to kill host cells to its advantage since ribosome-inactivating proteins were previously considered as “suicidal agent,” exploited from plant to contain the spread of pathogens (Bonness et al., 1994; Park et al., 2004). Also, the viruses like Tobacco Mosaic Virus (TMV), *Turnip vein clearing virus* (TVCV), *Cauliflower mosaic virus*, and *Chinese wheat mosaic virus* can exploit the interaction between a own movement protein and a PME for cell-to-cell movement (Chen et al., 2000; Chen and Citovsky, 2003). PME-dependent formation of methanol and PME-dependent enhancement of RNA silencing also influences viral cell-to-cell movement (Dorokhov et al., 2006, 2012). A tobacco PMEI (FN432040) is a methanol-inducible gene involved in defense reactions. The overexpression of AtPMEI2 in Arabidopsis and AcPMEI in tobacco contrasts the cell-to-cell and systemic movement of tobamoviruses (Lionetti et al., 2014b, 2015a).

The ability of plants to sense and maintain pectin integrity is important for salt tolerance (Yan et al., 2018; Liu et al., 2021). A fine control of different PME isoforms could modulate the ion-binding capacities of CWs to cope with salt stress (Pilling et al., 2004). AtPMEI17 positively contributes to salt tolerance in Arabidopsis (Chen et al., 2018; Liu et al., 2021). *AtPMEI17* overexpression showed decreased PME activity, increased pectin methylesterification, and an improved seeds germination, root growth and survival rate under salt stress compared to control. Instead, AtPMEI10 is a negative regulator of salinity tolerance (Jithesh et al., 2012). *Atpmei10* mutants upon NaCl treatment showed enhanced root growth and biomass yield and a reduced salt stress.

The ubiquitous AtPMEI-PME3 was also involved in basal metal tolerance to Zinc (Weber et al., 2013). A defective proteolytic cleavage of PMEI domain from catalytic part of AtPMEI-PME3 cluster *in ozs2* (overly zinc sensitive 2) mutant, causes a root hypersensitivity to zinc. The PME activity, by producing free carboxylic groups, could potentially favor the binding of metal cations to CW thereby lowering their uptake into the symplast. Similarly, AtPMEI-PME46 mediated de-methylesterification reduces aluminum binding to CWs and hence alleviating aluminum-induced root growth inhibition (Geng et al., 2017).

Pectin contents, PME activity, and pectin methylesterification are dynamically regulated during plant acclimation to temperature stresses (Solecka et al., 2008; Baldwin et al., 2014). Under chilling stress PME activity can increase the stiffness of CWs, increasing cold and freezing tolerance for the plant (Qu et al., 2011). AtPMEI-PME41 is proposed to modulate the chilling tolerance by modifying the mechanical properties of CW though the brassinosteroid signaling. AtPMEI17 (named AtPMEI13 in the original article) negatively contributes to Arabidopsis freezing tolerance (Chen et al., 2018). However, AtPMEI17 overexpressors showed longer roots under less severe cold conditions, suggesting a role of this inhibitor in balancing the trade-off between freezing tolerance and growth maintenance under low-temperature conditions. AtPMEI-PME34 can regulate the rate of transpiration during the heat response (Huang et al., 2017; Wu et al., 2017). This cluster is highly expressed in guard cell where it contributes to regulate CW flexibility and heat tolerance, promoting stomatal movement.

## INV Inhibitors

The first INVI was identified and biochemically characterized in potato (Schwimmer et al., 1961). Later, INVI isoforms were also identified in tobacco, maize (*Zea mays*), tomato, potato, soybean (*glycine max*), and Arabidopsis (Greiner et al., 1998; Bate et al., 2004; Reca et al., 2008; Liu et al., 2010; Su et al., 2016; Tang et al., 2017). To date, only two INVI/PMEI genes were annotated to encode INVIs in *A. thaliana.* These genes, originally termed AtC/VIF1 and AtC/VIF2, were cloned in *E. coli*, their activity characterized *in vitro* and identified as INVIs (Link et al., 2004). AtC/VIF1 exhibited an apoplastic localization and inhibited a large proportion of CW-INV activity in Arabidopsis (Su et al., 2016). The situation is less clear for AtC/VIF2, which inhibited both V-INV and CW-INV activity, but the affinity for V-INV activity was about 10-fold higher than that for CW-INV activity (Link et al., 2004). However, AtC/VIF2 clearly localized in the cell wall. To standardize the nomenclature of the INVI/PMEI superfamily and enjoying a broader view of information, we rename these two proteins as AtCW-INVI1 and AtCW/V-INVI2, respectively. Unlike PMEIs, the physiological information on INVIs in Arabidopsis is scarce. The contribution to understanding their roles comes mainly from experiments carried out in other plant species (Figures 5, 6).

### INVIs Affect Seed Germination, Seedling Growth, Senescence, and Sugar Content in Fruits

Some INVIs can regulate cell elongation and division. In Arabidopsis, AtCW-INVI1 can influence sugar metabolism and transport in seeds and roots. *atcw-invi1* mutant showed a faster seed germination and an increased root length in seedlings compared to control (Su et al., 2016). CW-INV activity can support cell division of the endosperm and embryo during early kernel development (Bate et al., 2004). Zm-INVINH1 is a maize CW-INVI that localizes specifically to the embryo surrounding regions. Zm-INVINH1 activity could compartmentalize invertase activity within the early kernel to allow the endosperm and embryo to follow distinct developmental programs.

CW-INVs play roles also in seed filling and fruit set in a wide range of plant species (Zanor et al., 2009; Wang and Ruan, 2012; Li et al., 2013; Liao et al., 2020). The control of INV activity by CW-INVIs is part of sugar unloading from phloem to fruits. In tomato, a knock-down expression of the apoplastic SlINVINH1 by RNAi technology caused an improvement in seed filling and sugar content in fruits (Jin et al., 2009). Later, a tomato knock-out for SlINVINH1 obtained using genome editing technology increases sugar content of tomato fruit without decrease fruit weight (Kawaguchi et al., 2021). Also in soybean, the suppression of the apoplastic *GmCIF1* improves seed weight (Tang et al., 2017). Also, fruit ripening seems affected by the balance between CW and V-INV activity. The repression of the vacuolar *SlVIF* by RNA interference delayed tomato fruit ripening and its overexpression increased ethylene production and led to a precocious color shift, due to elevated lycopene accumulation (Qin et al., 2016). Manipulation of INVI-INV interactions represents a promising strategy to increase crop production and to produce crops with a high sugar content in fruits.

Leaf senescence is characterized by a nutrient relocation from leaves to other parts of the plant and the cytokinins delay senescence affecting source–sink relations (Guo et al., 2021). Different CW-INVIs were correlated to senescence. Silencing the *SlINVINH1* expression in tomato increases apoplastic INV activity and delays leaf senescence (Jin et al., 2009). Consistently, the expression of the INVI *P17A* in tobacco under control of a cytokinin-inducible promoter causes the loss of cytokinin-induced delay in senescence (Balibrea Lara et al., 2004).

### INVIs Play Roles During Biotic and Abiotic Stresses

Invertase activity and its post-translational modulation by INVIs are part of immune responses against microbes, especially in the apoplast (Tauzin and Giardina, 2014). The downregulation of AtCW/V-INVI2 expression and activity in Arabidopsis source leaves in response to infection by *P. syringae*, de-represses invertase activity as part of the plant defense response (Bonfig et al., 2010). However, invertase activity can also be exploited by pathogens in the root. Invertase gene expression is upregulated in root galls developed by *P. brassicae* in Arabidopsis (Siemens et al., 2011). The overproduction of AtCW-INVI1 and AtCW/V-INVI2 in Arabidopsis transgenic lines caused a reduced invertase activity in the root, a lower sucrose import into infected cells, leading to a reduced clubroot symptoms. The expressions of the two apoplastic *PtC/VIF1* and *PtC/VIF2* are strongly induced in the root of *Populus trichocarpa* against different stress cues including fusarium wilt (*Fusarium solani*), drought, abscisic acid, wound, and senescence (Su et al., 2020). A *Nicotiana attenuata* CW-INVI, named NaCWII, is strongly upregulated in a JA-dependent manner to increase secondary metabolite biosynthesis in *Manduca sexta*-attacked plants (Ferrieri et al., 2015).

Cold stress limits productivity and adversely affects plant growth and development. The content of sugars with an osmoprotective function increased during cold treatment (Janská et al., 2010). During chilling stress, tomato plants de-repress INV activity in the apoplast by controlling *SlINVINH1*expression (Xu et al., 2017). Cold storage of potato tubers prevent sprouting and pathogenesis favoring the maintenance of supply throughout the year. However, cold induces a breakdown of starch to sucrose that is ultimately cleaved into glucose and fructose by acid invertases, leading to a tuber sweetening (Mckenzie et al., 2013). Cold-induced sweetening is a serious postharvest problem for potato compromising tuber quality. The ectopic expression of different V-INVIs in potato tubers prevents cold-induced sweetening by capping the activities of V-INVs (Greiner et al., 1999; Brummell et al., 2011; Liu et al., 2013; Mckenzie et al., 2013).

Stomatal movement is critical in plant response to drought and V-INV activity is correlated with stomatal aperture under normal and drought conditions (Ni, 2012). The ectopic expression of the tobacco V-INVI, *Nt-inhh*, under the control of an ABA-sensitive and guard cell-specific promoter AtRab18 conferred enhanced drought tolerance in Arabidopsis and tomato (Chen et al., 2016). More recently, the drought-responsive apoplastic Zm-INVINH4 was identified and characterized in maize (Chen et al., 2019). Moreover, Arabidopsis salt tolerance can be influenced by At*CW-INVI1* (Yang et al., 2020). Transgenic plant overexpressing At*CW-INVI1* showed enhanced sensitivity to ABA and reduced tolerance to salt.

## Undefined INVI/PMEIs and Their Possible Roles

Some knowledge was collected for INVI/PMEI members from now on presented. However, new experiments are needed to define their PMEI or INVI activity and to elucidate their roles in plant physiology. A INVI/PMEI isoforms, named AppB1 (At4g24640) was previously associated to pollen development (Holmes-Davis et al., 2005). A large-scale mutant screen in *A. thaliana* led to the identification of two INVI/PMEI mutants with defects in female gametophyte development and function (Pagnussat et al., 2005). The mutant embryo sac development arrest 24 (EDA 24) fails in polar nuclei fusion during the embryo sac development while the mutant unfertilized embryo sac 11 (UNE11) is affected in embryo sac fertilization. At5g46950 (named InvINH2) is an endosperm-specific INVI-PMEI, but its specific activity remain still to be characterized (Zuma et al., 2018). A genome wide association study supported by quantitative trait loci mapping identified the INVI/PMEI At1g23350 as a candidate gene for the response to drought/*B. cinerea* sequential double-stress combination (Coolen et al., 2019).

## Conclusion and Perspective

Data obtained on the pattern of expression, specific activity, and related physiological effects deepen our knowledge of INVI/PMEI roles in plant growth and defense and allow to engineer precise biotechnological applications. A stage-specific manipulation of INVI/PMEIs *in planta* could be used as biotechnological strategy to control the fruit growth and postharvest fruit softening. INVI/PMEI represent also genetic sources to generate crop varieties, either by traditional breeding or by genetic engineering, with a durable resistance to stresses and/or with a high crop yield. The revision allowed to eliminate various inaccuracies in the nomenclature of INVI/PMEI members, providing a clear tool for future studies. We emphasize the importance of verifying the inhibitory activity of new members of the superfamily not yet characterized, before assigning them an identity. Some shortcomings on the family certainly emerge, especially on the physiological role of INVIs, and several questions remain unanswered. Although a vacuolar, cytosolic or apoplastic function was suggested for some INVI/PMEI isoforms, further investigation is needed to understand their subcellular action and processing. Future research could also try to reveal the dynamics of inhibition as well as the fate of the inhibitors after performing their function. Moreover, PMEIs were linked to the modulation of the degree and pattern of methylesterification of HG. However, an interesting and not yet tested hypothesis is that some PME and PMEI isoforms could be dedicated to xylogalacturonan or rhamnogalacturonans, which are other pectic polysaccharides showing some degree of methylesterification. PMEIs, like also other apoplastic factor as pH and calcium concentration could influence not only the degree but also the pattern of methylesterification. Studies aimed at identifying the three-dimensional structure of PMEI-PMEs could provide important information useful to clarify the role of PMEIs in these clusters. New knowledge on the interactions between specific PMEI and PME isoforms and on the inhibition features in PMEI-PMEs isoforms will be necessary to understand the dynamics of the control of PME activity in plant physiology.

## Author Contributions

DC and VL collected data from literature and prepared figures and revised the paper. VL designed and wrote the manuscript. All authors contributed to the article and approved the submitted version.

## Funding

The work was supported by Sapienza University of Rome, Grants RM120172B78CFDF2 and RM11916B7A142CF1 to VL and AR12117A8A4A1ADC to DC and LV.

## Conflict of Interest

The authors declare that the research was conducted in the absence of any commercial or financial relationships that could be construed as a potential conflict of interest.

## Publisher’s Note

All claims expressed in this article are solely those of the authors and do not necessarily represent those of their affiliated organizations, or those of the publisher, the editors and the reviewers. Any product that may be evaluated in this article, or claim that may be made by its manufacturer, is not guaranteed or endorsed by the publisher.

## Footnotes

^1^ http://pfam.xfam.org/family/PF04043

^2^ www.cazy.org

## Abbreviations

Ac: *Actinidia chinensis*
At: *Arabidopsis thaliana*
CIF1: Cell Wall Inhibitor of *β*-Fructosidase
C/VIFs: Cell Wall/Vacuolar Inhibitor of β-Fructosidases
CW: Cell Wall
CWDEs: Cell Wall Degrading Enzymes
CW-INV: Cell Wall Invertase
CW-INVI: Cell Wall Invertase Inhibitor
EDA: embryo sac development arrest
ERF: Ethylene Responsive Element Binding Factor
FER: FERONIA
GH: Glycoside Hydrolase family
GL: GLABRA
Gm: *Glycine max*
HG: Homogalacturonan
HMS: Highly Methyl Esterified Seeds
INV: Invertase
INVI: Invertase Inhibitor
ISR: Induced Systemic Resistance
JA: Jasmonic Acid
LUH/MUM: Leunig Homolog/Mucilage Modified
Ma: *Musa acuminata*
MYB: Myeloblastosis
*ozs2*: overly Zinc sensitive 2
PME: Pectin Methylesterase
PMEI: Pectin Methylesterase Inhibitor
Sl: *Solanum lycopercsicum*
STK: Seedstick
TMV: Tobacco Mosaic Virus
TVCV: Turnip Vein Clearing Virus
UNE: Unfertilized Embryo Sac
V-INV: Vacuolar Invertase
Vv: *Vitis vinifera*
WAK: Wall Associated Kinase
Zm: *Zea mays*
